# Prevalence of Fear of Falling and Its Association With Physical Function and Fall History Among Senior Citizens Living in Rural Areas of China

**DOI:** 10.3389/fpubh.2021.766959

**Published:** 2021-12-23

**Authors:** Qingqing Su, Yuan Gao, Jie Zhang, Jingping Tang, Mi Song, Jie Song, Yazhan Mao, Hongying Pi

**Affiliations:** ^1^Medical School of Chinese PLA, Beijing, China; ^2^Department of Nursing, The First Medical Center, Chinese PLA General Hospital, Beijing, China; ^3^Department of Nursing, The Sixth Medical Center, Chinese PLA General Hospital, Beijing, China; ^4^Department of Rheumatology, The First Medical Center, Chinese PLA General Hospital, Beijing, China; ^5^Medical Service Training Center, Chinese PLA General Hospital, Beijing, China

**Keywords:** fear of falling (FOF), accidental fall, senior citizens, physical functional performance, rural

## Abstract

**Background:** Fear of falling (FOF) is as significant as a fall, leading to limited physical activity and poor quality of life among senior citizens. This study aimed to investigate the prevalence of FOF and its association with physical function and fall history among the senior citizens (≥75 years old) living in rural areas of China.

**Methods:** This was a cross-sectional study conducted in eastern China from June to October 2019. All elderly participants were recruited during their attendance for the free health examinations in villages and towns organized by the local healthcare authorities. Data on sociodemographics, fall history, FOF conditions, self-reported comorbidity and regular medications were collected by face-to-face interview, and the physical function status was evaluated through a field test. Univariate and multivariate analyses were performed to compare the differences in physical function and fall history of senior citizens with/without FOF.

**Results:** A total of 753 senior citizens (mean age = 79.04) participated in this study. Of these, 63.5% were aged 75–80. FOF was reported in 22.8% of the participants, while 18.5% had a fall in the past year. Among the senior citizens with and without a fall history, the prevalences of FOF were 38.8 and 19.2%, respectively. On multivariate analyses, FOF was independently associated with the Time Up and Go Test (TUG) duration (OR = 1.080; 95% CI: 1.034–1.128), 4-Stage Balance Test score (OR = 0.746; 95% CI: 0.597–0.931), fall history (OR = 2.633; 95% CI: 1.742–3.980), cerebral apoplexy (OR = 2.478; 95% CI: 1.276–4.813) and comorbidities (≥2) (OR = 1.637; 95% CI: 1.066–2.514), while the correlation between FOF and the 30-s chair stand test was only statistically significant in univariate analysis (*Z* = −3.528, *p* < 0.001).

**Conclusion:** High prevalence of FOF is observed among the senior citizens living in rural areas of China. FOF is strongly correlated with physical function performance and fall history. Therefore, the implementation of targeted FOF prevention measures is key to improve the physical activity of the senior citizens, which would ultimately lead to fall prevention and improved quality of life.

## Introduction

Fall is a global public health concern given the aging population worldwide (1). It may negatively impact individual physical and mental health, potentially leading to functional decline, disability, and premature death (2, 3). Fall-related psychological disorders mainly include fear of falling (FOF), loss of self-efficacy, and avoidance of certain activities, which threaten the overall health of senior citizens (4, 5). In particular, FOF is considered equally as important as falls in the elderly and demands effective management (6). Investigations on the FOF shed light on further understanding of falls in the elderly. Compared with falls that occur only at a time point, FOF as an outcome variable provides superior consistency of research. As a psychological phenomenon and without intervention, FOF is unlikely to decrease but persist and progress over time (7). With differences in the definitions and methods of FOF measurement used in various studies and the population objects, the prevalence of FOF of senior citizens has been reported to range widely from 3 to 85% (8, 9).

FOF is defined as ongoing concern about falls, which may result in an individual avoiding daily living tasks (one or more) that he/she is otherwise capable of due to continuous attention to fall issues (10). Activities restricted by such concerns are usually closely related to fall risk, a common and serious complication among the elderly (11). FOF is attributed to many factors. It has been initially described as a post-fall syndrome, suggesting its association with previous fall experience (12). However, studies have found that senior citizens with a fall history have a FOF prevalence of 40–73%, while half of those without a fall history have also reported FOF (13). Hence, senior citizens may be exposed to FOF regardless of their fall histories (8). In addition, some scholars have acknowledged the need to assess the physical function (such as balance, gait, muscle strength, etc.) along with the psychological support in older individuals, given that FOF is expected to decrease with improvements in physical performance (6, 14). Besides fall history and physical function, factors including gender, age, medication history, comorbidity, etc., may also contribute to FOF in the elderly (15, 16). The falls efficacy scale or a single question “Are you afraid of falling?” is a generally accepted tool used by researchers to measure FOF (17, 18).

Previous studies have mainly focused on the impact of a single physical function performance on FOF, and involved participants who are primarily elderly over 60 years old in urban communities or those suffering from a specific disease. To date, data on FOF and the physical function of the elderly living in rural areas of China are scarce. Given that the living facilities and medical security in rural areas are more deficient when compared with those in urban areas, the elderly in rural areas may have a higher risk of falling and the incidence of FOF, which deserves our attention. Our study aimed to evaluate the prevalence of FOF and its association with physical function and fall history among senior citizens (>75 years old) living in rural areas of China, which would provide a valuable reference for the future screening, prevention, and treatment of FOF and its associated adverse outcomes.

## Materials and Methods

### Study Design and Participants

This cross-sectional study was conducted on the basis of an annual free physical examination for the elderly in the township organized by the Kunshan Health Commission of Jiangsu Province and undertaken by the Physical examination Center of Jinxi People's Hospital. All the elderly who participated in physical examination came from the Jinxi town and its 20 administrative villages. Senior citizens undergoing physical examination from June to October 2019 were recruited. The inclusion criteria were aged 75 years or older, living in the local area for more than 10 years, able to communicate verbally, and able to walk independently (with the walking aids was allowed). Senior citizens diagnosed with dementia or with Mini-Mental State Examination (MMSE) score ≤24 (19) and who had attended the emergency room or been hospitalization within 3 months were excluded. A total of 753 senior citizens were recruited in the study, with the mean MMSE score of participants of 28.61 (1.01). Written informed consent was obtained from all the participants. This study was approved by the medical ethics committee of Chinese PLA General Hospital (Approval No.: S2018-048-01).

### Data Collection

Data on socio-demographics and comorbidity of participants, including age, gender, residence status, body mass index (BMI), self-reported medical illness (hypertension, diabetes, cerebral apoplexy, osteoporosis, arthritis and urinary incontinence) and types of medication, FOF and falls were obtained by face-to-face interviews. Data on physical function (balance, gait and muscle strength) was evaluated through a field test. Surveys were conducted by experienced medical personnel who regularly engaged in geriatric care and had received formal training before the survey. All investigators were divided into two groups, those responsible for collecting physical function data or other data. Investigators who collected the physical function data were blinded to the FOF results.

### Fear of Falling

All participants were asked a single question, “Are you afraid of falling”? When the participant answered “yes,” we would consider he or she had FOF. This single-item question had a simple structure and was easy to implement and manage, even in individuals with cognitive impairment. Therefore, it has been widely used and considered a gold standard evaluation (7). Previous studies have revealed that the evaluation outcomes of the single question were equivalent to that of the Fall Efficacy Scale and Fall Efficacy Scale–International (FES-I) (18, 20). For identifying FOF in the population, the re-test result of using the single question within 2 weeks was reliable (*kappa* = 0.72) (21).

### Physical Function

The Time Up and Go (TUG) test was used to evaluate the dynamic balance of the body, the activity of lower limbs and gait characteristics (22). Studies have shown that the TUG test has good sensitivity (*Sen* = 91%) and specificity (*Spe* = 82%), which is a reliable tool to identify fall risk in senior citizens (23, 24). During the test, participants were required to wear comfortable shoes. To begin, the participant was required to sit on a standard seat with a seat height of 43 cm and an arm height of 21 cm. When the participant heard the “start” command, he/she got up and walked 3 meters away with normal steps, turned back to the seat and sat down. The time (seconds) of the whole process and any abnormal gaits during walking were recorded, including slow tentative pace, loss of balance, short strides, little or no arm swing, steadying self on walls, shuffling, and bloc turning (25). Upon standing up or sitting down, participants were allowed to be supported by their arms. During walking, participants were also allowed to use walking aids if required. If walking aids were used, the appropriate and correct use of the equipment was recorded. Each participant was required to complete three repeats of the TUG test.

The 4-stage balance test was performed to evaluate the static balance, which required the participant to complete four standing postures with a gradual increase in difficulty, including (a) standing with feet close together and side by side; (b) standing with feet close together and half in series; (c) standing with feet in series (heel-toe); (d) standing on one foot (26). Each posture was considered complete and awarded 1 point if the participant successfully maintained the posture for 10 s without support or help. Otherwise, 0 points were awarded. The total score ranged from 0 to 4 points. A high score indicated a good balance (27). Several authors have reported that the test has excellent test-retest (*r* = 0.97) and inter-evaluator reliabilities (kappa = 0.92) (17).

The 30-s chair stand test was performed to measure the lower limb muscle endurance, which has been verified to have a high test-retest correlation and good criterion-related validity in both males and females (*r* = 0.78 in men and *r* = 0.71 in women) (28). To begin, the participant was required to sit on a chair with a seat height of 43 cm and no arms, holding his/her arms across the chest and the feet were shoulder-width apart. When the “start” command was heard, the participant quickly stood up from the chair, stood completely straight, and then quickly sit down (25). Participants were requested to complete the “stand up-sit down” action as swiftly as possible, and the number of complete actions within 30 s was recorded.

### Falls

The unified question: “Have you ever fallen in the past 12 months?” was asked to the participants to determine any incidence of fall in the past year. A “yes” to the question indicated a positive fall history or otherwise. A fall was defined as an unintentional fall to the ground, floor, or lower level (29).

### Statistical Analysis

Statistical analyses were performed by using the SPSS 24.0 statistical software. Categorical variables were described by frequency and percentage. Continuous variables with normal distribution were described by mean and standard deviation, while non-normal distribution data were expressed by median and quartile. Bivariate analyses were performed using the Chi-square test for categorical variables and the Mann-Whitney *U*-test for continuous variables. Finally, taking the FOF as the dependent variable, variables with a statistically significant difference in the univariate analysis were determined as the independent variables and included in the binary Logistic regression model to explore the correlations of FOF with physical activity and fall history in senior citizens. A *P*-value of <0.05 was considered statistically significant.

## Results

A total of 753 senior citizens were recruited in the study. The mean age of participants was 79.04 (3.66) years, and most belonged to the group aged from 75 to 80 (63.5%). Of all participants, 45.4% were female, 17.5% lived alone, 55.2% had BMI within the normal range (18.5–23.9), 58.4% suffered from hypertension, 7.3% suffered from diabetes, 6.9% had cerebral apoplexy, 9.8% were diagnosed to have osteoporosis, 18.2% had arthritis, 1.6% reported to have urinary incontinence, 24.2% had 2 or more chronic diseases, and 3.3% took ≥5 regular medications. There were 172 (22.8%) participants who reported having FOF, and 139 (18.5%) had at least one fall in the past year.

The proportions of individuals with FOF in the 80–85-year-old (29.1%) and over 85-year-old groups (26.1%) were higher than that in the 75–80-year-old group (19.7%) (Table 1). Meanwhile, the prevalence of FOF in females was higher than that of males (26.3 vs. 20.0%). When compared with healthy senior citizens, those with hypertension, cerebral apoplexy, or urinary incontinence had a higher prevalence of FOF, and senior citizens with FOF were more likely to have two or more comorbidities. Furthermore, senior citizens taking≥5 regular medications had a higher proportion of individuals with FOF than those taking less than five.

**Table 1:** Comparisons of socio-demographic characteristics and comorbidities between participants with and without FOF.

**Variables**	**Total samples** **(*n* = 753)**	**With FOF** **(*n* = 172)**	**Without FOF** **(*n* = 581)**	***p*-value**
Age (years), *n* (%)				0.021^*^
75–80	478 (63.5)	94 (19.7)	384 (80.3)	
80–85	206 (27.3)	60 (29.1)	146 (70.9)	
≥85	69 (9.2)	18 (26.1)	51 (73.9)	
Gender, *n* (%)				0.038^*^
Female	342 (45.4)	90 (26.3)	252 (73.7)	
Male	411 (54.6)	82 (20.0)	329 (80.0)	
Living alone, n (%)	132 (17.5)	33 (25.0)	99 (75.0)	0.515
BMI (kg/m^2^), *n* (%)				0.307
<18.5	66 (8.8)	13 (19.7)	53 (80.3)	
18.5–23.9	416 (55.2)	87 (20.9)	329 (79.1)	
24–27.9	223 (29.6)	58 (26.0)	165 (74.0)	
≥28	48 (6.4)	14 (29.2)	34 (70.8)	
Hypertension, *n* (%)	440 (58.4)	112 (25.5)	328 (74.5)	0.043^*^
Diabetes, *n* (%)	55 (7.3)	17 (30.9)	38 (69.1)	0.139
Cerebral apoplexy, *n* (%)	52 (6.9)	25 (48.1)	27 (51.9)	<0.001^**^
Osteoporosis, *n* (%)	74 (9.8)	22 (29.7)	52 (70.3)	0.137
Arthritis, *n* (%)	137 (18.2)	40 (29.2)	97 (70.8)	0.050
Urinary incontinence, *n* (%)	12 (1.6)	6 (50.0)	6 (50.0)	0.024^*^
Comorbidities (≥2), *n* (%)	182 (24.2)	62 (34.1)	120 (65.9)	<0.001^**^
Types of medication (≥5), *n* (%)	25 (3.3)	11 (44.0)	14 (56.0)	0.010^*^

*Comorbidities (≥2): Two or more medical illnesses (hypertension, diabetes, cerebral apoplexy, osteoporosis, arthritis and urinary incontinence). FOF, fear of falling; BMI, body mass index*.

* *p-value < 0.05*,

** *p-value < 0.01*.

The TUG tests revealed that senior citizens with FOF generally took a significantly longer time to complete the test than those without FOF (*Z* = −4.473, *p* < 0.001), showed more apparent gait abnormalities and required walking aids (Table 2). Moreover, senior citizens with FOF scored significantly lower in the 4-Stage Balance Test than those without FOF (*Z* = −3.882, *p* < 0.001). Furthermore, senior citizens with FOF completed a significantly lower number of the “stand up-sit down” actions in the 30-s chair stand test (*Z* = −3.528, *p* < 0.001). Additionally, FOF was more prevalent among senior citizens who had fallen in the past year (38.8%) than those with no fall history (19.2%).

**Table 2:** Correlation of FOF of senior citizens with physical function and fall history.

**Variables**	**Total samples** **(*n* = 753)**	**With FOF** **(*n* = 172)**	**Without FOF** **(*n* = 581)**	***p*-value**
TUG score, [Md(P_25_,P_75_)]	11.0 (9.0, 13.8)	12.0 (9.7, 15.8)	10.8 (8.8, 13.4)	<0.001^**^^†^
Abnormal gait, *n* (%)	485 (64.4)	134 (27.6)	351 (72.4)	<0.001^**^
Use walking aids, *n* (%)	24 (3.2)	12 (50.0)	12 (50.0)	0.001^*^
4-Stage Balance Test score, [Md(P_25_,P_75_)]	3.0 (2.0, 3.0)	3.0 (2.0, 3.0)	3.0 (2.0, 3.5)	<0.001^**^^†^
30-s chair stand test score [Md(P_25_,P_75_)]	11.0 (9.0, 14.0)	10.0 (9.0, 12.0)	12.0 (9.0, 14.0)	<0.001^**^^†^
Fall history, *n* (%)	139 (18.5)	54 (38.8)	85 (61.2)	<0.001^**^

*FOF, fear of falling; TUG, time up and go*.

† *Mann-Whitney U-test*.

* *p-value < 0.05*,

** *p-value < 0.01*.

### Correlation of FOF With Physical Function and Fall History

The multivariate regression analysis revealed that FOF was associated with times to complete the TUG test (OR = 1.080; 95% CI: 1.034–1.128), 4-Stage Balance Test scores (OR = 0.746; 95% CI: 0.597–0.931), fall history (OR = 2.633; 95% CI: 1.742–3.980), cerebral apoplexy (OR = 2.478; 95% CI: 1.276–4.813) and two or more comorbidities (OR = 1.637; 95% CI: 1.066–2.514) (Table 3).

**Table 3:** Multivariate regression analysis of variables associated with FOF among senior citizens.

**Independent variables**	**B**	**S.E**.	**Wald**	***p*-value**	**OR (95% CI)**
TUG score	0.077	0.022	12.119	<0.001^**^	1.080 (1.034–1.128)
4–Stage Balance Test score	−0.293	0.113	6.699	0.010^*^	0.746 (0.597–0.931)
Fall history	0.968	0.211	21.104	<0.001^**^	2.633 (1.742–3.980)
Cerebral apoplexy	0.908	0.339	7.182	0.007^*^	2.478 (1.276–4.813)
Comorbidities (≥2)	0.493	0.219	5.062	0.024^*^	1.637 (1.066–2.514)
Constants	−1.785	0.486	13.482	<0.001^**^	0.168

*Comorbidities (≥2): two or more medical illnesses (hypertension, diabetes, cerebral apoplexy, osteoporosis, arthritis and urinary incontinence). FOF, fear of falling; TUG, time up and go*.

* *p-value < 0.05*,

** *p-value < 0.01*.

## Discussion

To date, few studies have investigated FOF in senior citizens, especially those over 75 years old living in rural areas of China. As a result of significant disparity between health policies implemented in the urban and rural areas by the central and local governments, health-related concerns among senior citizens living in rural areas have somewhat been overlooked (30). Notably, the rural residents account for nearly 50% of the total population (~605,990,000) in China. Therefore, it is essential to evaluate FOF in senior citizens living in rural areas of China, whereby our findings provided an intuitive understanding of FOF and its relationship with physical functions in geriatrics, which may be helpful for fall risk management of the elderly in the rural communities.

The prevalence of FOF in senior citizens varies by population. A cross-sectional study on FOF among senior citizens aged over 65 in Hong Kong, China has shown that 64.7% of the participants had FOF, while 65.6% of the participants had no fall history (31). Another study from China has found the FOF prevalence of 81.0% in senior citizens of urban communities (32). In our study, the overall FOF prevalence was 22.8%, while the FOF prevalences among senior citizens with and without fall history were 38.8 and 19.2%, respectively, which was far lower than those reported in previous studies. The low FOF prevalence in our study might be attributed to our participants mainly consisted of farmers who work all year round demanding intense ability in physical activity and reasonable confidence that they will not fall. Consistent with our study, a Thai study on the FOF among the elderly in suburban and semi-rural areas has also reported a relatively low incidence of FOF, 25.2%, with most participants of the study being farmers, who were adapted to life working in paddy fields and wetlands (33). Moreover, our participants were senior citizens undergoing physical health checks in the hospital, who were relatively active and healthy, and thus had a higher fall efficacy (34). Nevertheless, further studies would be conducted to explore the difference in FOF among senior citizens from rural and urban communities.

The association between decreased physical function and FOF is multifaceted and multidirectional (35). Reduced physical performance may lead to a degree of fear of falling (36), while individuals with FOF may avoid physical activities, resulting in a decline in physical function (37). Our findings revealed that senior citizens with FOF not only scored significantly lower in the 4-Stage Balance Test but also required significantly longer time to complete the TUG test, accountable for a higher proportion of individuals with abnormal gait and using walking aids. These findings were consistent with the study by Hoang et al. (38) and Kalinowski et al. (39), indicating that senior citizens with FOF are more inclined to have a relatively poor balance and walking ability. Although our multivariate analysis did not demonstrate an independent association of FOF with gait abnormalities and walking aids usage, other variables including the time taken to complete the TUG test and 4-Stage Balance Test score were independent predictors of FOF. These suggest that regardless of fall history, a decreased physical performance may lead to reduced ability to respond to physical challenges (such as adaptability to challenges in physical balance), which might increase the fear of falls, and vice versa. Conversely, FOF may also directly affect physical function performance (40, 41). Previous studies have demonstrated the association of FOF with lower limb muscle strength (42). Senior citizens may reduce their daily activities due to FOF, resulting in a further decrease in muscle strength. Perpetually, as a result of muscle weakness, they may be unable to perform a routine daily activity that further weakens their muscle strength, leading to the loss of confidence in completing daily tasks without falling (43). However, although our univariate analyses demonstrated a significant correlation between lower limb muscle strength and FOF, this correlation was not evident in the multivariate analysis, which is consistent with the study by Khalil et al. (44). These disparities in findings from different studies are likely attributed to variability in population characteristics or living environments. Despite the cross-sectional nature of our study and the relative time of decreased physical function and FOF being elusive, their interaction remains undebatable.

It has been observed that community-based tai chi, home-based exercises, and multifactorial physical intervention toward minimizing falls at home have reduced FOF rates (45). Perhaps, a well-planned exercise and rehabilitation program applied with the cohort in rural areas may lessen FOF and fall rates, consequently increasing their quality of life, which will be conducive to the realization of active aging.

Our study showed that fall history was the key influencing factor of FOF in senior citizens (>75 years old) living in rural areas of China. Moreover, senior citizens with a fall history had 2.6 folds higher prevalence of FOF than those with no fall history, which was consistent with the findings of previous studies. Earlier studies on the association between fall history and FOF have focused on older individuals in healthy communities or with specific diseases, with little research on rural senior citizens (14, 46). Thus, our study has contributed to the completeness of this data. Moreover, it also reminds us of the obligation to investigate the fall history during the health examination. However, more recent studies have reported that senior citizens with no fall history are also exposed to severe FOF (13). An 11-year longitudinal study of incident events in older individuals has found that the history of falls at baseline did not predict acquiring a FOF, nor did FOF predict a fall later (47). Despite inconsistencies regarding the correlation between FOF and fall history, most studies have provided supporting evidence on the universality of FOF in senior citizens. Our study has highlighted that the preventative intervention for FOF should be instituted on senior citizens with a fall history in the past year. Meanwhile, those with no history of falls but have deteriorating physical function may also benefit from such intervention. Additionally, consistent with findings of previous studies (41, 48), our analyses also revealed that factors including cerebral apoplexy and two or more comorbidities were independently associated with FOF. Expectantly, senior citizens with cerebral apoplexy or multiple comorbidities would likely be in poor health and lacking in physical ability, leading to FOF.

There were limitations to our study. Firstly, our cross-sectional study did not allow for analyses of causality, which should be explored in future longitudinal studies of older populations living in rural areas. Secondly, significant selection bias might have occurred in our study. Given that all participants in our study came from one physical examination center and did not show substantial cognitive impairment, our results might not be generalisable to all rural senior citizens. Thirdly, recall bias might have been introduced in our data collection, and the daily physical activities of the elderly and some other potential confounders (such as types of medication, visual acuity, hearing ability, etc.) were not collected. Thus, future research should expand the included factors to further clarify the relationship between FOF and physical function.

## Conclusions

FOF among senior citizens living in rural areas of China has a robust independent relationship with physical function and fall history. Early identification and management of FOF are vital to prevent further deterioration in physical function. On the other hand, improving the adaptability of senior citizens to challenges in physical balance, enhancing autonomy in physical activity, and fall prevention are effective strategies to curb FOF, which ultimately leads to improvements in the quality of life of senior citizens.

## Data Availability Statement

The raw data supporting the conclusions of this article will be made available by the authors, without undue reservation.

## Ethics Statement

The studies involving human participants were reviewed and approved by Chinese PLA General Hospital Medical Ethics Committee. The patients/participants provided their written informed consent to participate in this study.

## Author Contributions

QS conceived the idea with HP, collected the data and did the data analysis. HP supervised the project. QS drafted the manuscript. All authors contributed to data analysis and manuscript revising.

## Funding

This work was supported by the National Key Research and Development Program of China (2018YFC2001400).

## Conflict of Interest

The authors declare that the research was conducted in the absence of any commercial or financial relationships that could be construed as a potential conflict of interest.

## Publisher's Note

All claims expressed in this article are solely those of the authors and do not necessarily represent those of their affiliated organizations, or those of the publisher, the editors and the reviewers. Any product that may be evaluated in this article, or claim that may be made by its manufacturer, is not guaranteed or endorsed by the publisher.
